# Innate Immunity in Autoimmune Thyroid Disease during Pregnancy

**DOI:** 10.3390/ijms242015442

**Published:** 2023-10-22

**Authors:** Tatjana Bogović Crnčić, Neva Girotto, Maja Ilić Tomaš, Ines Krištofić, Sanja Klobučar, Lara Batičić, Božena Ćurko-Cofek, Vlatka Sotošek

**Affiliations:** 1 Department of Nuclear Medicine, Faculty of Medicine, University of Rijeka, Braće Branchetta 20, 51000 Rijeka, Croatia; tatjanabc@uniri.hr (T.B.C.); maja.ilic.tomas@uniri.hr (M.I.T.); 2 Department of Obstetrics and Gynecology, Faculty of Medicine, University of Rijeka, Braće Branchetta 20, 51000 Rijeka, Croatia; ines.kristofic@uniri.hr; 3 Department of Internal Medicine, Faculty of Medicine, University of Rijeka, Braće Branchetta 20, 51000 Rijeka, Croatia; sanja.klobucar@uniri.hr; 4 Department of Medical Chemistry, Biochemistry and Clinical Chemistry, Faculty of Medicine, University of Rijeka, Braće Branchetta 20, 51000 Rijeka, Croatia; lara.baticic@uniri.hr; 5 Department of Physiology, Immunology and Pathophysiology, Faculty of Medicine, University of Rijeka, Braće Branchetta 20, 51000 Rijeka, Croatia; bozena.curko.cofek@uniri.hr; 6 Department of Anesthesiology, Reanimatology, Emergency and Intensive Care Medicine, University of Rijeka, Braće Branchetta 20, 51000 Rijeka, Croatia; vlatkast@uniri.hr; 7 Department of Clinical Medical Sciences II, Faculty of Health Studies, University of Rijeka, Viktora Cara Emina 2, 51000 Rijeka, Croatia

**Keywords:** autoimmune thyroid disease, pregnancy, neutrophils, dendritic cells, NK cells, NKT cells

## Abstract

Autoimmune thyroid disease (AITD) is the most common organ-specific autoimmune disorder clinically presented as Hashimoto thyroiditis (HT) and Graves’ disease (GD). The pathogenesis of AITD is caused by an inappropriate immune response related to genetic, non-genetic, and environmental factors. Pregnancy is one of the factors that have a great influence on the function of the thyroid gland because of the increased metabolic demand and the effects of hormones related to pregnancy. During pregnancy, an adaptation of the maternal immune system occurs, especially of the innate immune system engaged in maintaining adaptive immunity in the tolerant state, preventing the rejection of the fetus. Pregnancy-related hormonal changes (estrogen, progesterone, hCG) may modulate the activity of innate immune cells, potentially worsening the course of AITD during pregnancy. This especially applies to NK cells, which are associated with exacerbation of HD and GD. On the other hand, previous thyroid disorders can affect fertility and cause adverse outcomes of pregnancy, such as placental abruption, spontaneous abortion, and premature delivery. Additionally, it can cause fetal growth retardation and may contribute to impaired neuropsychological development of the fetus. Therefore, maintaining the thyroid equilibrium in women of reproductive age and in pregnant women is of the highest importance.

## 1. Introduction

Autoimmune thyroid disease (AITD) is the most common organ-specific autoimmune disorder presenting clinically as Hashimoto thyroiditis (HT) and Graves’ disease (GD). Estimates suggest that AITD affects considerably more women than men, with a prevalence of 5–15% vs. 1–5%, respectively [1]. In addition, an increase in the incidence of AITD has been reported in recent years [2,3]. The underlying pathophysiology of AITD includes an aberrant immune response targeted against own body antigens in thyroid cells due to the lack of self-tolerance, in interaction with genetic, non-genetic, and environmental factors. 

Genetic factors connected with AITD include polymorphism in human leukocyte antigen system (HLA) genes, cytotoxic T-lymphocyte-associated protein 4 (CTLA-4) gene, protein tyrosine phosphatase non-receptor type 22 (PTPN22) gene, FOXP3 gene [4], chromosome X inactivation patterns, cytokines and receptors interleukin-2 (IL-2), IL-2 receptor (IL-2R), estrogen receptors, adhesion molecules (CD14, CD40), selenoprotein S, and gene products associated with apoptosis. There are also thyroid-specific AITD susceptibility genes, such as thyroid-stimulating hormone receptor (TSHR), thyroglobulin (Tg), and thyreoperoxidase (TPO) genes, potentially connected to AITD [4]. Since the risk of AITD for some of these genes is rather low, it is considered that some epigenetic factors might be necessary to develop AITD, such as methylation, histone modification, and RNA interference [5,6,7].

Non-genetic, environmental factors usually have a role in genetically predisposed patients and include cigarette smoking, alcohol, exposure to radiation and chemicals (phthalates, flame retardants), bacterial and viral infections (*Yersinia enterocolitica*, *Helicobacter pylori*, hepatitis C virus), but the risk has also been described with living in sterile conditions. Dietary problems connected with higher AITD prevalence are iron deficiency, decreased selenium and vitamin D intake, and a gluten-rich diet. Variable iodine exposure with higher AITD prevalence in iodine-rich and lower prevalence in iodine-deficient areas is already well known [8,9]. Gut microbiota with *Bacteroides fragilis* overgrowth, stress, and certain drugs, such as tyrosine kinase inhibitors, amiodarone, and lithium are also considered risk factors for AITD development [10,11].

Clinical presentation of overt AITD is generally the reflection of the hormonal status, with some exceptions in subclinical cases. Although GD and HT are also functionally and clinically different entities, they are pathophysiologically related and may cross-develop. In the case of HT, which is characterized by an increase in thyrotropin (TSH) and lower levels of thyroid hormones, triiodothyronine (T3), and thyroxine (T4), the signs of thyroid hypofunction prevail, including weight gain, slower heart rate, slow bowel movement with constipation, enlarged thyroid gland, normocytic anemia, skin changes, hair and body hair loss, edema, fatigue, and memory impairment [12]. On the other hand, hyperthyroidism in GD is characterized by the opposite, higher T3 and T4 hormone levels, suppressed TSH, weight loss, rapid heartbeat, and accelerated bowel movement with diarrhea, while thyroid gland enlargement, hair loss, and fatigue are also present but caused by different underlying pathophysiological mechanisms. There are also effects connected with the reproductive system, such as anovulatory cycles due to impaired estrogen precursor conversion and menorrhagia [13]. Subclinical cases of HT are defined as elevated TSH with normal thyroid hormone levels, and although the majority is asymptomatic, there are some clinical data on increased rate of cardiovascular morbidity in those patients [14,15,16].

In patients with initial HT, symptoms and signs of thyroid hyperfunction can be seen due to enhanced thyroid hormone release from damaged thyroid cells into circulation. This type of thyrotoxicosis is self-limiting and further progresses to permanent hypothyroidism. In silent and postpartum thyroiditis, thyrotoxicosis is also present but is of shorter duration, followed by hypothyroidism, which in the majority of cases recovers completely within the first year postpartum. 

Apart from genetic and environmental factors, pregnancy has a great influence on the function of the thyroid gland. It has been proposed that there is an export of cells between the mother and the fetus in both directions, starting from the second week of pregnancy, including cells of immune origin, which can persist in the host for years, and is referred to as fetal and maternal cell microchimerism [17].

For about 20 weeks, the fetus is dependent on the maternal production of thyroid hormones. After that, the fetal thyroid begins to produce its own hormones but is still dependent on the mother’s iodine supply. Thyroid volume increases by 10% to 40% depending on iodine status. Therefore, an iodine intake of 250 micrograms/day is recommended during pregnancy, and 150 micrograms/day is required for women planning pregnancy or breastfeeding [18].

To meet the increased metabolic demand during pregnancy, there are significant changes in thyroid function, such as an increase in serum thyroid-binding globulin (TBG) and stimulation of TSHR by human chorionic gonadotropin (hCG). 

Serum TBG concentrations during pregnancy rise nearly twofold because estrogen increases TBG production and TBG sialylation, which results in a decreased clearance of TBG [18,19]. 

hCG stimulates the secretion of thyroid hormones and transiently suppresses the maternal TSH, especially in the first trimester in 15% of healthy pregnant women [18]. This is known as gestational transient thyrotoxicosis. There are changes in the reference values of maternal TSH; the lower limit decreases for 0.1–0.2 mU/L and the upper limit for 0.5–1.0 mU/L compared with the reference range of non-pregnant women [20], which is especially pronounced in multiple pregnancies. Circulating TBG and total thyroxine (TT4) concentrations gradually increase until 16 weeks of gestation and remain high until delivery [18].

In addition, pregnancy can trigger the onset of AITD, leading to autoimmune hyperthyroidism or autoimmune hypothyroidism and affecting the course of pregnancy. 

## 2. The Delicate Thyroid Equilibrium in Pregnancy

Thyroid dysfunction is a pathological condition commonly seen in pregnant women, and likewise, AITD can undesirably affect fertility. Its association with adverse outcomes such as placental abruption, spontaneous abortion, and premature delivery [21], gestational hypertension, and fetal growth retardation is confirmed by a plethora of studies [22]. Furthermore, thyroid misbalance can contribute to impaired neuropsychological development of the fetus [23,24]. A large recent quantitative study showed that increased autoantibodies to TPO (TPOAb) and thyroglobulin (TGAb) levels were related to a higher probability of pregnancy-induced hypertension, while both TPOAb and TGAb levels were negatively correlated with neonatal birth weight [25]. 

Maternal biochemical hypothyroidism is usually a priori defined as TSH above 6.0 mIU/L, and exposure to TSH above 10 mIU/L is used as a marker of overt maternal hypothyroidism. It is known that subclinical thyroid dysfunction before conception is related to an increased risk of adverse pregnancy outcomes [26], which significantly occur in pregnant women having TSH above 10 mIU/L. Interestingly, no significant association between thyroid autoantibodies and pregnancy outcomes was found in recent research by Knøsgaard et al. [27]. Increasing evidence emphasizes an elevated risk of adverse gestational and neonatal outcomes induced by subclinical hypothyroidism in women during the early gestational period. Particularly, the risk of pregnancy-specific complications has been increased in TPOAb-positive women having serum TSH levels exceeding 2.5 mIU/L, especially in those with serum TSH levels greater than 4.0 mIU/L [28]. Unfortunately, results of randomized clinical trials revealed that thyroid hormone supplementation is unsuccessful in the prevention of adverse pregnancy outcomes in women with AITD. However, Negro et al. reported that selenium supplementation during pregnancy and in the postpartum period can reduce thyroid inflammation and consequently the development of hypothyroidism [29] Therefore, future research and scientific efforts should be focused on the possibilities of immunotherapies [22]. 

Concerns were raised about thyroid dysfunction in newborns whose mothers have (autoimmune) hypothyroidism, which led to the practice of thyroid hormone testing in the early neonatal period. However, a recent large observational study by Perez et al. revealed that infants born from women with (autoimmune) hypothyroidism had normal thyroid function in the early neonatal period. Just 1% of infants were detected with TSH levels > 10 mIU/L that normalized after four weeks. Therefore, it was concluded that routine thyroid hormone testing in addition to newborn screening tests does not bring additional benefits [30]. 

## 3. Autoimmune Hyperthyroidism in Pregnancy

The most common cause of hyperthyroidism in pregnancy is GD, which occurs in about 0.2% of all pregnancies in iodine-rich areas [20,31], whereas in iodine-deficient countries, excessive thyroid hormone production is usually caused by autonomous thyroid nodules [32], GD often improves during the third trimester of pregnancy and may worsen during the first year after birth. It is characterized by the production of thyroid-stimulating immunoglobulin (TSI) autoantibodies that bind to the TSH receptor and stimulate the overproduction of thyroid hormones. TSI antibodies cross the placenta and cause fetal or neonatal hyperthyroidism (incidence between 1% and 5% in women with GD) [33]. However, if the mother is taking antithyroid drugs (ATDs) for GD, fetal hyperthyroidism is rare because ATDs also cross the placenta, preventing fetal hyperthyroidism [34]. Elevated TPOAb or TgAb were found in 2% to 17% of pregnant women [35]. They also cross the placenta but are not associated with fetal thyroid dysfunction. Elevated levels of autoantibodies increase the risk of thyroid dysfunction in the postpartum period and GD may then worsen, although the most common cause of thyrotoxicosis in this period is postpartum thyroiditis with a prevalence of 4% [36]. 

It is very difficult to distinguish GD from gestational transient thyrotoxicosis (GTT) in early pregnancy since both conditions have similar symptoms: palpitations, tremors, and heat intolerance. The likelihood of transient thyrotoxicosis during pregnancy is higher in pregnant women who have not had previous thyroid disease, goiter, or orbitopathy and have a mild vomiting disorder [37]. 

Hyperthyroidism in pregnancy can also be caused by toxic multinodular goiter and toxic adenoma [38]. Subacute painful or silent thyroiditis are very rare causes of thyrotoxicosis in pregnancy, while TSH-secreting pituitary adenoma, struma ovarii, functional thyroid cancer metastases, or a TSH receptor mutation leading to functional hypersensitivity to hCG [39] are even rarer. 

Poor control of thyrotoxicosis is associated with pre-eclampsia, pregnancy loss, prematurity, low birth weight, intrauterine growth restriction, and thyroid storm [40]. 

## 4. Autoimmune Hypothyroidism in Pregnancy 

Hypothyroidism in pregnancy is commonly defined as the presence of elevated TSH and decreased serum T4 concentration during gestation, with both concentrations outside the trimester-specific reference ranges. For women with a TSH above the population and trimester-specific upper limit, or above 4.0 mIU/L when local reference ranges are not available, free T4 should be measured [41]. The most common cause of thyroid hypofunction during pregnancy is HT.

It is estimated that 2–3% of healthy non-pregnant women of reproductive age have elevated TSH, and thyroid autoantibodies are present in around 30–60% of those cases. [42].

Continuing pregnancies with overt hypothyroidism have been associated with an increased risk of several complications such as preeclampsia, gestational hypertension, placental abruption, preterm delivery, low birth weight, increased rate of cesarean section, postpartum hemorrhage, perinatal morbidity and mortality, and neuropsychological and cognitive impairment in the child. 

De Leo reports the presence of TPOAbs and TgAbs in 5–14% and 3–18% of pregnant women, respectively [43]. In addition to this, TPOAbs were found in 9.5% of women with previous pregnancy loss or subfertility [44].

AITD is the most common autoimmune disease in women of reproductive age, affecting nearly 10% of that group. Keeping this in mind is of great interest to clinicians because of the potential negative impact on female fertility and pregnancy outcomes [45].

The exact mechanism is far from being fully understood, so the aim of this review is to describe the pathophysiology of AITD during pregnancy, focusing on the role of innate immunity.

## 5. The Immunological Background of AITD

The immunological background of AITD includes innate and adaptive mechanisms. In general, innate mechanisms are cell-mediated and ensure a prompt response to pathogens, whereas adaptive mechanisms are specific and involve the formation of antibodies targeted to specific antigens [46] but also seem to be directed by innate factors. HT is primarily characterized by cell-mediated autoimmunity in the form of lymphocytic infiltration, causing the destruction of thyroid follicles. It has recently been proposed that the presence of specific stromal cells in the thyroid of patients with HT stimulates inflammatory cell recruitment, even the formation of highly organized lymphocytic infiltrates termed tertiary lymphoid organs [47]. Tissue destruction and the consequent exposure of thyroid antigens cause autoantibody production. In GD, the lymphocytic infiltration is milder, and the immune processes are predominantly humoral [48,49], but the two mechanisms are closely connected [50]. 

Contrary to adaptive immunity that has been extensively described in AITD, the cells of innate immunity involved in the pathophysiology of AITD have recently gained more attention. The main cells involved in the innate immunological processes are polymorphonuclear leukocytes (mainly neutrophils), innate lymphoid cells including natural killer (NK) cells, natural killer T (NKT) cells, monocytes, macrophages, and dendritic cells (DCs) [51,52,53,54]. The activation of cellular immune mechanisms generally involves cytokine release, lymphocytic infiltration, and cell destruction [55]. This part of immunity is generally considered non-specific, but some specific elements to it have recently been described [56]. 

## 6. Innate Immunity during Normal Pregnancy 

**Figure 1:**
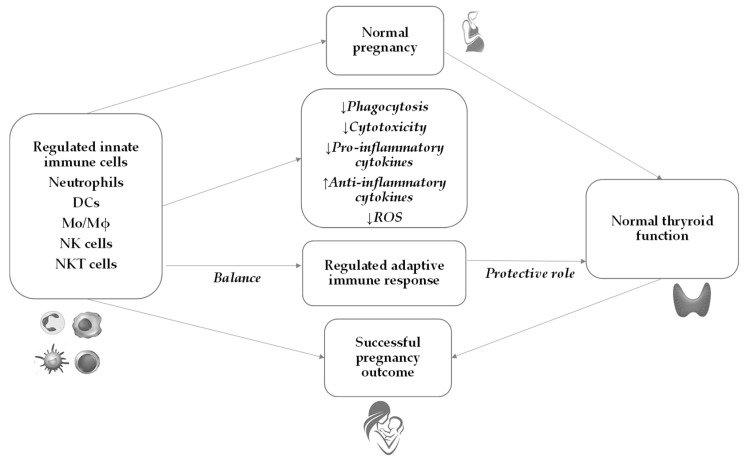
Pregnancy outcome in normal pregnancy. In a normal pregnancy, a well-regulated innate immune response leads to balanced thyroid function and contributes to a successful pregnancy outcome. DCs—dendritic cells; Mo—monocytes; Mϕ—macrophages; ROS—reactive oxygen species; NK—natural killer; NKT—natural killer T cells.

During normal pregnancy, a complex and extensive adaptation of the maternal immune system occurs to protect the mother and the fetus from infection and activation of detrimental immune response against the semi-allogeneic fetus. Throughout pregnancy, the innate immune system changes and plays a critical role in maintaining adaptive immunity in the tolerant state (Figure 1). This tolerogenic state during pregnancy is well established systemically and especially locally where maternal and fetal tissues are in direct contact with each other. It is considered that decidua and, as lately described, placenta play pivotal roles in the innate immune response [57].

Throughout pregnancy, the decidual cells secrete chemokines that attract NK cells, NKT cells, DCs, macrophages, and T-regulatory (Tregs) lymphocytes. During the initial weeks of pregnancy, about 70% of decidual lymphocytes are NK cells, 20% are macrophages, and only 2% are DCs. In contrast to decidual tissue, up to 90% of maternal peripheral blood leukocytes are comprised of neutrophils [58,59,60].

This increased number of neutrophils during pregnancy, especially in the second and third trimesters, is called neutrophilia and is a physiological condition [60]. The neutrophils are considered the first line of innate immunity responsible for the defense of the mother and the fetus due to their ability to exert a variety of mechanisms to combat tissue damage and infection such as cytotoxic activity, phagocytosis, synthesis of reactive nitrogen species (RNS), reactive oxygen species (ROS), and secretion of a variety of cytokines and chemokines [61,62]. 

Additionally, neutrophils interact with macrophages, DCs, NK cells, and T and B lymphocytes modulating innate and adaptive immune responses [63]. Besides in peripheral blood, neutrophils are present in decidua, predominantly in decidua basalis, where they have an important role during implantation and trophoblast migration [57]. 

Together with an increased number of neutrophils, the number of monocytes increases during pregnancy, starting in the first trimester [64]. This upregulation of monocytes is predominantly due to a higher proportion of intermediate CD14^+^CD16^+^ monocytes, which are responsible for pro-inflammatory cytokine production, such as interleukin (IL)-1α, IL-6, IL-12, tumor necrosis factor-alpha (TNF-α) [65] and decreased phagocytic activity throughout pregnancy [66]. An increased number of monocytes is accompanied by their progressive activation [67]. The monocytes display increased levels of activated markers such as CD11a, CD11b, and CD14 as well as higher ROS and inflammatory cytokine production [68]. The exact mechanism of monocyte activation is unknown, but it seems that placenta-derived extracellular vesicles can induce monocyte activation and maturation [69]. 

In decidua, macrophages represent the second most abundant decidual leukocytes after fertilization, attracted by decidual-derived chemokines such as monocyte chemoattractant protein-1 (MCP-1), MCP-3, macrophage inflammatory protein 1-alpha (MIP-1α), MIP-1β, MIP-2γ, and fractalkine [70]. There are two different types of macrophages, M1 with pro-inflammatory functions and M2 with immunomodulatory functions [71]. Inflammatory M1 macrophages are associated with implantation, followed by an increase in the M2 phenotype, which plays an important role in maintaining pregnancy [72]. Decidual macrophages play an important role in the control of spiral artery remodeling, promote endometrial angiogenesis, and modulate the inflammatory environment and trophoblast invasion [57]. 

Besides neutrophils and monocytes/macrophages, it is suggested that DCs also contribute to successful pregnancy [73]. Decidual DCs are an immunotolerant and heterogenous population that modulate vascular expression and angiogenesis during implantation [74]. After implantation, the number of DCs progressively declines [75]. DCs closely interact with other cells such as macrophages, NK cells, and T cells to keep the appropriate environment for pregnancy maintenance [74]. 

The most predominant and the best-characterized cells of innate immunity throughout pregnancy are NK cells [76]. It is proposed that peripheral blood NK cells under the influence of cytokines (predominantly IL-15) and hormones (progesterone) proliferate, migrate to the decidua, and differentiate into decidual NK cells by transforming growth factor-beta (TGF-β) and IL-11 [57]. The decidual NK cells are gaining much attention because their number increases dramatically in the first trimester of pregnancy, reaching over 70% of all decidual lymphocytes [77]. They appear to be beneficial for placental development, control of trophoblast invasion, and angiogenesis [77,78]. The low cytotoxic activity of decidual NK cells is the result of the expression of the natural killer group (NKG) 2 receptor, which recognizes HLA ligands (HLA-E, HLA-C, and HLA-C) and supports fetomaternal tolerance [79]. 

## 7. Innate Immunity in AITD and Pregnancy 

**Figure 2:**
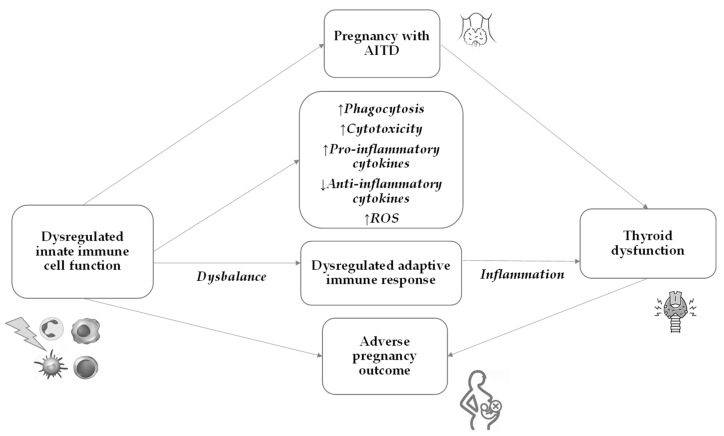
Pregnancy outcome in pregnancy with AITD. In pregnancy with AITD, dysregulated innate immune cell function leads to a dysregulated adaptive immune response, which causes thyroid dysfunction and may lead to an adverse pregnancy outcome. AITD—autoimmune thyroid disease; ROS—reactive oxygen species.

The innate immune system plays a pivotal role in the early recognition and initiation of the immune response against the thyroid gland in AITD. The subsequent activation of the adaptive immune response further perpetuates the autoimmune process, leading to thyroid dysfunction (Figure 2).

The role of immune cells, including macrophages, neutrophils, and DCs, is essential in both the development of AITD and its impact on pregnancy (Table 1). The immune system undergoes significant changes during pregnancy to prevent the fetus from being rejected as a foreign body. Pregnancy induces a shift toward a more tolerant immune response to accommodate the fetus [80]. However, the dysregulated immune response in AITD may affect maternal immune adaptations during pregnancy, potentially influencing the risk of pregnancy complications.

### 7.1. Dendritic Cells

AITD occurs, in part, because of changes in DC function as a result of genetic and environmental alterations. Disruption of the tolerance network contributed by each DC subset can promote autoreactive T-cell responses and pathology [75,81].

DCs are responsible for capturing, processing, and presenting thyroid antigens (such as the TSHR) to other immune cells, primarily T cells, in the nearby lymph nodes. This process activates T cells specific to thyroid antigens and triggers the autoimmune response against the thyroid gland. DCs can also influence the balance between pro-inflammatory (Th1 and Th17) and anti-inflammatory (regulatory T cells or Tregs) responses. A disbalance in this T-cell response can contribute to the development or progression of AITD [50].

Moreover, DCs located at the maternal–fetal interface interact closely with other components of the immune system to maintain an optimal balance for physiological pregnancy outcomes [82]. Decidual DCs are faced with two challenges: they must avoid activating T cells specific for inoffensive antigens or risk-inducing autoimmune disease while also being alert to pathogens so that they can prime T cells when needed. This polarity is amplified during pregnancy since DCs must protect against infection while also being compliant with the novel antigens expressed by the placenta [83].

Pregnancy causes significant hormonal changes, including an increase in estrogen, progesterone, and glucocorticoid levels, which can affect the immune system. These hormonal changes may modulate the activity of innate immune cells, potentially affecting the course of autoimmune thyroid disease during pregnancy. There is evidence that estrogen can activate DCs, whereas progesterone and glucocorticoids inhibit their action [84,85].

### 7.2. Macrophages

Macrophages are important phagocytes and antigen-presenting cells (APCs) with the main function to phagocytose danger signals such as cell fragments and pathogens in the body and to present antigens to adaptive immune cells to produce subsequent immune responses [86]. It is widely accepted that the overactivation of M1 macrophages can mistakenly present autoantigens to T cells and B cells, causing the body to produce autoantibodies or autoreactive lymphocytes, thus triggering autoimmune diseases. At the same time, M1 macrophages can also mediate the occurrence of autoimmune diseases by producing various pro-inflammatory factors. Multiple studies have shown that abnormal polarization of macrophages is closely related to the occurrence and progression of various autoimmune diseases, including AITD [87,88,89].

Macrophages play an important role in the immune response underlying AITD pathogenesis by being involved in several key processes such as inflammation, antigen presentation and cytokine production [90]. 

As potent antigen-presenting cells, macrophages process and present antigens from the thyroid gland to other immune cells, including T cells, thus initiating or exacerbating the autoimmune response against the thyroid. In HT, the thyroid gland is infiltrated by macrophages, leading to chronic inflammation. Inflammation leads to the destruction of thyroid cells and ultimately the impairment of thyroid function, causing hypothyroidism. In addition, activated macrophages can produce pro-inflammatory cytokines, such as IL-1, IL-6, and TNF-α, which further promote the recruitment and activation of other immune cells and contribute to the ongoing inflammatory process. Macrophages can additionally contribute to the destruction of thyroid tissue by producing ROS [91,92].

### 7.3. Neutrophils

Neutrophils are another type of innate immune cells involved in the innate inflammatory response. In AITD, particularly in GD, the thyroid gland can be infiltrated by neutrophils, which contribute to local inflammation and tissue damage. Neutrophils in AITD can also release neutrophil extracellular traps (NETs), which are web-like structures composed of DNA and antimicrobial proteins. These NETs can promote autoimmunity by triggering the activation of immune cells and exacerbating inflammation in the thyroid [93].

There are limited data on the role of macrophages and neutrophils in autoimmune thyroid disease during pregnancy. Future studies are needed to shed more light on the interplay between different cells of innate immunity and their function in the pathogenesis of AITD during pregnancy. 

### 7.4. NK Cells

NK cells are an essential component of innate immunity and, together with T lymphocytes, constitute the executive effectors of cell-mediated immunity. They account for 10–15% of lymphocytes in peripheral blood [94]. The majority of NK cells (90%) in peripheral blood are CD56^dim^ and are described as predominantly cytotoxic, whereas the minority are CD56^bright^ and are thought to be responsible for the production of immunomodulatory cytokines [95]. NK cells are considered regulators of acquired immunity because they provide stimulatory signals to T cells and dendritic cells. This interaction confers a role in numerous immune responses and makes them participants in various diseases such as infections, cancer, and autoimmune diseases [96]. The main characteristic of NK cells is their ability to kill targets (virus-infected or malignant cells) without prior sensitization or coupling with HLA molecules, unlike T lymphocytes. They are activated by the pro-inflammatory cytokines IL-2, interferon (IFN)-γ, IFN-β, and IL-12 [94]. While IL-2 induces NK cell proliferation and cytotoxicity, IFN-γ only enhances NK cytolytic activity [97].

Research has shown that NK cell activity is regulated by the expression of NK receptors on the cell surface, which can be divided into activating and inhibitory [98]. These cells have a very sophisticated program to control activation and killing, and it is now known that this involves a complex system of various inhibitory and activating receptor-ligand interactions in conjunction with changes in HLA expression. Inhibitory receptors stop activating signal transduction, whereas binding of a specific ligand to the activating NK receptor leads to NK cell activation and initiates the executive mechanisms of cytotoxicity and cytokine production [98]. There are two main mechanisms by which NK cells kill: the first one is by the direct release of cytotoxic granules (perforin and the granzyme family) into the immune synapse and the second one is by binding to the so-called “death receptors” on the target cells (TNF receptor family), the cytolytic Fas/FasL mechanism being one of the most important [99]. In the resting state, NK cells express few cytokines, but when activated, they can secrete numerous cytokines, including IFN-γ, TNF-α, and TNF-β [100]. TNF-α and IFN-γ produced by activated NK cells are thought to be responsible for the induction of apoptosis and are involved in the control of the immune response [101].

Because of their cytolytic activity, production of cytokines, and interaction with antigen-presenting cells, T cells, and B cells, NK cells are thought to play an important role in the pathogenesis of AITD. Studies have shown that NK cell numbers are reduced, and their cytotoxic capacity is impaired in most autoimmune diseases [102]. It has been suggested that a viral antigen is responsible for disturbances in NK cell activity in autoimmune diseases such as multiple sclerosis and Crohn’s disease [103] and that autoimmunity may be related to chronic viral infections due to suppression of NK cell cytotoxicity against virus-infected cells or due to NK modulation of the immune response by regulating the expansion of B and T cells [97].

However, there are different views regarding NK activity in AITD. Although the exact mechanism of immunoendocrine interactions is still unknown, the existence of receptors for thyrotropic and thyroid hormones on lymphocytes has been described and TSH production by human immune cells after stimulation with staphylococcal enterotoxin A and thyrotropin-releasing hormone (TRH) has been demonstrated [97]. These results suggest that the immune system is involved in the regulation of thyroid hormone metabolism. In addition, the expression of TSHR was found in lymphoid and myeloid cells [104]. TSH in combination with IL-2 has a co-stimulatory effect on NK cells. 

It is also known that the pro-inflammatory Th1 cytokines IL-2, TNF-α, and IFN-γ are involved in the development of autoimmunity, including AITD [97]. Some authors suggest that NK cells may play a regulatory role in autoimmunity by actively suppressing the generation of the autoreactive immune response [102]. Since NK cells are the source of Th2 cytokines, their decreased number may lead to the dominance of the Th1 cytokine profile and the development of autoimmunity [96]. Hence, it has been suggested that NK cells are associated with exacerbation of HD and GD [105]. 

Cell destruction is thought to be mediated by cellular mechanisms such as autoreactive T lymphocytes, NK cells, and cytokines. Solerte et al. [106] noted that alterations in NK cell cytotoxicity and cytokine production occur in both HT and GD, and that normalization of NK function helps prevent the onset and progression of both diseases. 

According to Ciampolillo et al., patients with AITD have significantly lower NK and CD25^+^ cell levels than healthy controls [107]. However, other authors note that the decreased effector activity of peripheral blood lymphocytes from patients with hyperthyroidism is likely due to a functional defect rather than a decreased number of NK cells and that incubation of peripheral blood lymphocytes from patients with GD with recombinant human pro-inflammatory IL-2 cytokine rapidly reverses this functional deficit [98]. However, the results are contradictory because the measurement of NK activity in the peripheral blood of patients with GD using a cytotoxicity assay or phenotypic analysis has yielded different results in different studies. Some investigators demonstrated increased NK cell activity in both HT and GD [108], while others observed normal NK activity [8]. It has also been suggested that thyrotoxicosis stimulates NK activity since an increased level of NK-activating cytokine IL-12 was found in GD [109]. 

Corrales et al. [110] emphasized that cytolytic activity decreases in patients with hyperthyroidism, which may be due to the metabolic effect of thyroid hormones on NK cell number and function. In accordance with the above, it seems that a disbalance in NK cell activity associated with an altered cytokine network may be one of the triggering factors for the occurrence of AITD. Specific changes in the number and activity of NK cells are also present in pregnant women with AITD. It is well known that human pregnancy is characterized by a complex set of antigen-specific and non-specific immunological changes that prevent fetal semi-allograft rejection [111], and this “suppressive” milieu is abruptly interrupted after delivery. 

Several immune mechanisms are thought to contribute to maternal immune tolerance during pregnancy. During normal pregnancy, both the activity and percentage of NK cells in the peripheral blood tend to increase in the first trimester and then decrease in the second trimester, with a further decrease in the third trimester [112]. In contrast, the increased number and killer activity of NK cells have been shown to increase the risk of miscarriage and to be associated with recurrent miscarriages [96]. The decline in peripheral blood NK cells has been found to be mainly due to the decline in the CD16^+^ subpopulation. In addition, NK cells from the peripheral blood of healthy pregnant women have lower lytic activity compared with controls [113]. Increased expression of inhibitory receptors (various KIR receptors, including CD94/NKG2A) was also found on NK and T cells in the first trimester of pregnancy, with the highest levels in the third month of pregnancy and a gradual decrease to baseline levels at the end of pregnancy [114]. Kung et al. also observed a decrease in the total number of T lymphocytes, T-helper lymphocytes, and NK cells but not B cells in pregnant women with GD. Indeed, pregnant women with GD had significantly more CD5^+^B cells than controls at all stages of pregnancy [35]. It seems that NK cells are involved in the immunosuppressive state of normal pregnancy and different NK subsets contribute to these changes [115].

In addition, increased NK activity was found during postpartum exacerbation of AITD [98]. These data may suggest that an increase in NK activity is associated with the worsening of AITD in HT and GD and that NK cells play an important role in the progression of AITD. 

More than 95% of all decidual CD56^bright^ cells express cytolytic mediator perforin, so the perforin content in the decidua is higher than in any other tissue under physiological and pathophysiological conditions [116]. The cytolytic activity of unstimulated decidual lymphocytes mediated by perforin has been shown to be higher than that of lymphocytes from the peripheral blood of pregnant women, and it has been described that decidual lymphocytes can utilize the cytolytic mediator Fas/FasL in addition to perforin [117]. Despite numerous research works, the exact role and reason for this efficient killing mechanism of NK cells on the maternal-fetal surface are still unknown. NK cells are thought to play a role in stimulating decidualization, placental and trophoblast growth, and immunomodulation, i.e., in shaping the acquired immune response and regulating homeostasis because of their ability to secrete a substantial amount of cytokines, as well as in defense against various pathogenic microorganisms [118]. Namely, mRNAs for TNF-α, IFN-γ, TGF-ß, granulocyte colony-stimulating factor (G-CSF), macrophage colony-stimulating factor (M-CSF), granulocyte-macrophage colony-stimulating factor (GM-CSF), and leukemia inhibitory factor have been found in decidual NK cells, while only mRNAs of TGF-β1 and TNF-α have been detected in the resting peripheral blood NK cells [119]. Significantly higher expression of CD69 on all subsets of NK cells (CD56^dim^, CD56^bright^, and CD16) was found in women with recurrent pregnancy loss, and the expression of the CD94/NKG2 inhibitory receptor was significantly decreased compared with controls [120].

The predominance of a pro-inflammatory cytokine milieu combined with changes in uterine NK cell number, phenotype, and function were proven to have an impact on reproductive outcomes. Therefore, the alterations in NK cell functions present in autoimmune thyroid disorders such as HT and GD could definitely play an important role in the course of a normal human pregnancy by altering appropriate cytokine support and immunomodulation, both in peripheral blood and maternal–fetal interface. 

### 7.5. NKT Cells 

NKT cells are a unique subpopulation of T lymphocytes that share the characteristics of both NK cells and conventional T lymphocytes and are involved in the regulation of complex immune responses associated with autoimmune diseases, infectious diseases, and cancer. Since NKT cells are activated at an early stage of the immune response and are thought to be responsible for numerous immunoregulatory activities, they are one of the key players determining the outcome of the immune response.

NKT cells form a small subpopulation of lymphocytes in the peripheral blood and were initially described as T lymphocytes because they express the semi-invariant T cell receptor (TCR). Like T cells, they are derived from thymic progenitor cells [121] but also have characteristics of NK cells such as CD161 or the NKR-P1 molecule.

There are three distinct populations of NKT cells: classical type I NKT (also known as invariant NKT, iNKT), type II NKT (non-classical), and NKT-like cells. Type I and type II are CD1d dependent, whereas NKT-like cells are CD1d independent. Type I NKT cells express a restricted type of TCR, while type II and especially type III NKT cells express a broader spectrum of TCR chains [122,123]. 

NKT cells support the adaptive T cell immune system by recognizing lipids but also contain preformed mRNA for cytokines, allowing rapid cytokine production, and are among the first responders in many infectious and inflammatory processes [121]. Thus, NKT cells influence both innate and adaptive immune responses. In addition, NKT cells express receptors for IL-12 and IL-18, making their cytokine receptor profile of innate immune cells [124]. In contrast to conventional T lymphocytes and Tregs, the T cell receptor of NKT cells does not react with peptide antigens presented by classical HLA molecules but recognizes glycolipids presented via CD1d, a non-classical antigen-presenting molecule within class I HLA molecules [123].

NKT cells preferentially secrete pro- or anti-inflammatory cytokines. However, because of the production of immunosuppressive cytokines such as IL-4, IL-10, and IL-13, these cells are thought to suppress the cellular immune response. Although NKT cells can potentially function as cellular effectors and exhibit cytotoxic activity, it is questionable whether all NKT cells have this capacity, and their actual physiological role is likely a regulatory function. This would be consistent with their small numbers, the regulatory effect of the cytokines they secrete, and the close interaction with dendritic cells and other cell types that influence acquired immunity [123].

The rapid response of NKT cells, indicating innate rather than acquired immunity, may allow NKT cells the regulation of acquired immune responses such as protection against viruses and bacteria and regulation of autoimmune diseases. In humans, only 0.2% of peripheral lymphocytes are NKT cells, yet these cells are thought to contribute to the maintenance of tolerance to autoantigens [125].

Human NKT cells are reduced in the peripheral blood of patients with various organ-specific and systemic autoimmune diseases but given the very low numbers and variability of NKT cells in human peripheral blood, evaluation of these cells is limited, which is why results may not be representative and must be interpreted with caution [126].

#### NKT Cells in Pregnant Women with AITD

The main pathogenic mechanism underlying the occurrence of AITD could be attributed to aberrant response or dysfunction of various immunoregulatory cell subsets triggered by environmental factors in genetically susceptible hosts [55,127]. 

A lower percentage of human NKT cells was found in the thyroid gland of patients with GD than in the peripheral blood of the same patients and in the peripheral blood of control subjects [128]. Roman-Gonzales et al. observed no significant difference in the frequency of iNKT cells in the peripheral blood of patients with HT and GD compared with the peripheral blood of control subjects [129]. They also found no significant differences in the production of pro- and anti-inflammatory cytokines by iNKT cells from patients with AITD.

NKT cells in first-trimester decidua account for 0.48% of CD3^+^ cells, which is 10-fold more than in peripheral blood. These cells produce IFN-γ and GM-CSF, whereas NKT cells from the peripheral blood of pregnant women secrete IL-4 [130]. Several studies suggest that iNKT cells may play a role in pregnancy. For example, treatment of pregnant mice with α-galcer results in pregnancy termination due to the secretion of IFN-γ by NKT cells, suggesting that intentional stimulation of NKT cells by glycolipids results in strong stimulation of NKT cells that do not necessarily have a tolerogenic effect. In addition, iNKT cells regulate NK cell activation and proliferation, and NK cells are critical for pregnancy progression and outcome [130,131]. Southcombe et al. examined the number and phenotype and the functional activity (production of IFN-γ) of iNKT cells in all trimesters in normal pregnancy and in pregnant women with preeclampsia as well as in controls. They found that the number and type of NKT cells did not differ during pregnancy, but the cells were activated, as evidenced by increased expression of CD69 on CD4/CD8 and CD8^+^ iNKT cells in the third trimester [132]. Decreased production of IFN-γ has also been described, consistent with the known shift to the anti-inflammatory phenotype of NK and NKT cells and the shift in cytokine secretion during pregnancy [132].

The exact role of NKT cells in pregnancy with AITD, both in peripheral blood and on the maternal-fetal surface, is still a mystery and yet to be discovered. Significantly elevated peripheral NK and NKT-like cell levels were found in euthyroid and subclinical hypothyroid women with thyroid autoimmunity and reproductive failure compared with healthy female controls. Within the NK population, it was found that the CD56^dim^ subset was increased and reproductive failure occurred more often. Still, there was no difference in the expression of different phenotypic markers by NK cells such as the activation marker (CD69), killer inhibitory receptor (CD158a, CD158b, ILT-2), and activating receptor (NKG2C, NKG2D) expression between euthyroid and subclinical hypothyroid women with thyroid autoimmunity and in healthy female controls. On the other hand, NKT-like cells in subclinical hypothyroid women with AITD and reproductive failure showed a significantly increased inhibitory CD158a receptor expression and a decreased level of activating NKG2D receptors, which could indicate an altered expression pattern of different NK cell activating and inhibitory receptors, resulting in functional abnormalities of NK and NKT-like cells [133]. The authors also confirmed enhanced cytotoxic activity by NK cells in the periphery of euthyroid and subclinical hypothyroid women with AITD and reproductive failure compared with healthy controls. In contrast, NKT-like cells did not show any changes in cytotoxic activity [133]. The results so far are not clear, but it seems that the dominance of pro-inflammatory immune response with dysbalanced NK and NKT-like cell ratios and cytotoxicity of women with thyroid autoimmunity could alter and negatively influence the pregnancy outcome.

**Table 1:** The most prominent changes in innate immune cells in patients with AITD, in women during normal pregnancy, and in pregnancy with AITD.

Cells of Innate Immunity	AITD		Normal Pregnancy	Pregnancy with AITD	
	HT	GD		HT	GD
Neutrophils	- increased number [93] - release extracellular traps [93]		- increased number, especially in 2nd and 3rd trimesters [60]	N/A	
Monocytes/Macrophages	- increased number of Mϕ in the thyroid gland [91] - Mϕ produce pro-inflammatory cytokines (IL-1, IL-6, TNF-α) [91] - Mϕ produce ROS [92]		- increased number of Mo from 1st trimester [64] - Mo activation [67,69] - Mo produce ROS and pro-inflammatory cytokines (IL-1, IL-6, and TNF-α) [65,68] - decreased Mϕ phagocytic activity [66] - Mϕ are the second most abundant decidual leukocytes [70]	N/A	
Dendritic cells	N/A		- decreased number after implantation [75]	N/A	
NK cells	- decreased number followed by exacerbation of HT and GD [105,107] - decreased production of Th2 cytokines [105] - increased number and cytotoxicity in women with AITD and reproductive failure [133]		- proliferation and migration to decidua [57] - increased number in decidua in 1st trimester [77] - increased number and activity in peripheral blood in 1st and decreased in 2nd and 3rd trimester [112] - decreased lytic activity [113] - cytokine secretion in dNK cells [118]	N/A	- decreased number [35]
NKT cells	- increased number in women with AITD and reproductive failure [133]	- lower number in the thyroid gland than in the peripheral blood [128]	- higher number in decidua than in the peripheral blood [130] - activated in 3rd trimester [132] - decreased production of IFN-γ [132]	N/A	

AITD—autoimmune thyroid disease; HT—Hashimoto thyroiditis; GD—Graves’ disease; IL—interleukin; Mo—monocytes; Mϕ—macrophages; N/A—not available; TNF-α—tumor necrosis factor-alpha; ROS—reactive oxygen species; IFN-γ—interferon gamma; NK—natural killer; dNK—decidual natural killer.

Although very little is known about their possible function, these classical NKT cells appear to be of great importance because they can mediate pro-inflammatory and anti-inflammatory responses and provide a “bridge” between innate and acquired immunity. It has been suggested that they may modulate the response of decidual NK cells and coordinate interactions between decidual leukocytes at the maternal–fetal interface [134]. 

The diverse role of NKT cells in various immunological conditions and diseases, particularly tumor immunity [135] and autoimmune processes [126], is now well documented, and there is increasing evidence that they also play a very important role in thyroid autoimmunity [133,136]. However, further studies are needed to fully understand the enigmatic mechanism by which NKT cells orchestrate.

## 8. Emerging Causal Connection: Microbiota and the Thyroid (Dis)balance

It is interesting to mention that studies analyzing the relationship between microbiota composition and thyroid function have been increasing rapidly in recent years. Evidence has been presented about the involvement of the gut microbiota in various aspects of thyroid pathology, which opened a new potential therapeutic target [137]. Since a healthy gut microbiota has beneficial effects on the activity of the immune system but also on thyroid function, the thyroid–gut axis emerged as a new focus in the field. The gut microbiota influences the availability of vital micronutrients: iodine, iron, and copper are crucial for thyroid hormone synthesis, selenium and zinc are required for converting T4 to T3, and vitamin D is involved in regulating the immune response. Those micronutrients are commonly deficient in AITDs, contributing to thyroid malfunctioning. Therefore, the interplay between gut microbiota and AITD should be taken into consideration when treating patients, especially pregnant women with thyroid diseases [138]. It has recently been found that gut microbiota exhibits distinctive appearances in patients with AITD, which could be associated with a disbalance in the immune system and gut microbiota [139]. Furthermore, a recent work by Calcaterra et al. suggests that supplementation with probiotics shows beneficial effects on thyroid function in pediatric patients as well since this approach could restore intestinal eubiosis and the proliferation of beneficial microorganisms [140]. 

## 9. Future Perspectives

The number of publications in the field of AITD increases every year, emphasizing the importance of this pathology, especially during pregnancy [141]. Medical research and improvements in diagnostics are constantly evolving. Therefore, future perspectives for AITD diagnostics in pregnancy rely on several aspects like the discovery of novel biomarkers specific to AITD in pregnancy. New biomarkers could support an earlier and more accurate diagnosis, allowing timely interventions to manage the thyroid condition effectively. In addition, improvements in imaging technologies might lead to better visualization of the thyroid gland and increase the possibility of detection of structural anomalies associated with AITD during pregnancy. More sophisticated ultrasound techniques or other imaging modalities could provide valuable insights into the structure of the thyroid gland affected by autoimmune processes. Furthermore, advancements in genetic screening might contribute to identifying women at higher risk of developing AITD during pregnancy, leading to targeted monitoring and early intervention in order to prevent possible issues. An improved understanding of individual distinctions in thyroid function and autoimmune responses might lead to progress in the personalized treatment of patients with AITD. Tailored treatments and interventions could optimize management for pregnant women affected by AITD. Future diagnostic approaches should involve the integration of multiple diagnostic tools and algorithms to provide a comprehensive assessment of thyroid health during pregnancy, taking into consideration different aspects such as hormone levels, imaging results, and medical history. However, it is important to emphasize that future perspectives are based on the potential direction of medical research and technological advancements. Target basic research on cell cultures and animal models as well as large clinical investigations are needed to contribute to the development of the field. Likewise, a growing impact of artificial intelligence and deep learning algorithms is expected to contribute to different aspects of detecting AITD. These new artificial tools hold great promise in improving the accuracy of AITD diagnosis [142]. 

## 10. Conclusions

The etiopathogenesis of AITD includes an immune reaction against own body antigens in thyroid cells of genetically susceptible individuals, in interaction with epigenetic and environmental factors. An activated cascade of immunological changes, which leads to the loss of immune tolerance, includes innate immunity, which further activates cells and mediators of adaptive immunity. Trigger antigen in this cascade as well as some other molecular events are still not known. For instance, a new area of investigation is the influence of gut dysbiosis on AITD. Until further studies resolve the background and help in new therapeutical approaches, it is of the utmost importance to identify and manage AITD in pregnant women to avoid adverse pregnancy outcomes and to ensure appropriate development of the newborns’ nervous system. Monitoring the thyroid function and proper thyroid hormone replacement medication are crucial to ensure that thyroid levels are within the appropriate range throughout the pregnancy. Regular follow-up visits and additional blood tests may be required to assess thyroid function and adjust medication dosage as needed. Proper diagnosis and management of autoimmune thyroiditis during pregnancy can significantly reduce the risk of complications for both the mother and the fetus. However, extensive studies are still needed to understand the complex immunopathogenesis of thyroid autoimmunity.

## Data Availability

Not applicable.
